# Prevention and Therapy of Hepatocellular Carcinoma by Vaccination with TM4SF5 Epitope-CpG-DNA-Liposome Complex without Carriers

**DOI:** 10.1371/journal.pone.0033121

**Published:** 2012-03-12

**Authors:** Sanghoon Kwon, Dongbum Kim, Byoung Kwon Park, Sunhee Cho, Kwang Dong Kim, Young-Eun Kim, Cheung-Seog Park, Hyun-Jong Ahn, Jae-Nam Seo, Kyung-Chan Choi, Doo-Sik Kim, Younghee Lee, Hyung-Joo Kwon

**Affiliations:** 1 Department of Microbiology, College of Medicine, Hallym University, Gangwon-do, Republic of Korea; 2 Division of Applied Life Science (BK21 Program), PMBBRC, Gyeongsang National University, Jinju, Republic of Korea; 3 Department of Biochemistry, College of Natural Sciences, Chungbuk National University, Chungbuk, Republic of Korea; 4 Department of Microbiology, College of Medicine, Kyung Hee University, Seoul, Republic of Korea; 5 Department of Pathology, College of Medicine, Hallym University, Gangwon-do, Republic of Korea; 6 Department of Biochemistry, College of Life Science and Biotechnology, Yonsei University, Seoul, Republic of Korea; 7 Center for Medical Science Research, College of Medicine, Hallym University, Gangwon-do, Republic of Korea; National Institutes of Health, United States of America

## Abstract

Although peptide vaccines have been actively studied in various animal models, their efficacy in treatment is limited. To improve the efficacy of peptide vaccines, we previously formulated an efficacious peptide vaccine without carriers using the natural phosphodiester bond CpG-DNA and a special liposome complex (Lipoplex(O)). Here, we show that immunization of mice with a complex consisting of peptide and Lipoplex(O) without carriers significantly induces peptide-specific IgG2a production in a CD4^+^ cells- and Th1 differentiation-dependent manner. The transmembrane 4 superfamily member 5 protein (TM4SF5) has gained attention as a target for hepatocellular carcinoma (HCC) therapy because it induces uncontrolled growth of human HCC cells via the loss of contact inhibition. Monoclonal antibodies specific to an epitope of human TM4SF5 (hTM4SF5R2-3) can recognize native mouse TM4SF5 and induce functional effects on mouse cancer cells. Pre-immunization with a complex of the hTM4SF5R2-3 epitope and Lipoplex(O) had prophylactic effects against tumor formation by HCC cells implanted in an mouse tumor model. Furthermore, therapeutic effects were revealed regarding the growth of HCC when the vaccine was injected into mice after tumor formation. These results suggest that our improved peptide vaccine technology provides a novel prophylaxis measure as well as therapy for HCC patients with TM4SF5-positive tumors.

## Introduction

Peptide vaccines are pivotal for inducing and regulating immune responses through their binding ability to the B cell receptor and MHC as B-cell epitopes and T-cell epitopes. Therefore, epitope-based peptide vaccines have gained attention as potentially useful prophylaxis for cancers and infectious diseases [1]–[4]. However, there is an important practical issue related to this: the limited efficacy of peptide vaccines in the treatment of humans. To improve the efficacy of peptide vaccines, liposomes have been evaluated for delivery of vaccines [5]–[8], and adjuvants such as flagella and CpG-DNA have been formulated to enhance the magnitude of the immune responses [9]–[12].

Liposomes have been extensively evaluated as vehicles for delivery in developing vaccines to enhance antibody production and cytotoxic T lymphocytes (CTL) responses [8], [13]–[15]. Encapsulated liposomes can protect antigens from the environment and deliver them to target cells. Cationic liposomes such as lipofectamin, 3β-[N-(N′,N′-dimethylaminoethane)-carbamoyl]cholesterol hydrochloride (DC-Chol), DC-Chol:phosphatidyl-β-oleoyl-γ-palmitoyl ethanolamine (DOPE), phosphatidylcholine:stearylamine:cholesterol have been shown to improve CTL response and antibody production [8]. In addition, pH-sensitive liposomes such as DOPE:cholesterol hemisuccinate (CHEMS) improve antigen delivery to the cytosol and the induction of CTL responses [14]. Sterically stabilized cationic liposomes such as DOPE:polyethylene glycol have also been used to increase the uptake of antigen in immune cells [15].

CpG-DNA, which contains unmethylated CpG dinucleotides flanked by specific base sequences and has immunostimulatory activities, has been investigated as a potentially useful prophylactic and therapeutic strategy [9], [16], [17]. CpG-DNA activates antigen-presenting cells such as dendritic cells and B cells, and induces Th1-biased immune responses and immunoglobulin (Ig) isotype switching [18]–[20]. The immunostimulatory activities of CpG-DNA as a potent adjuvant are enhanced by encapsulation in liposome [5], [21]. Several studies have indicated that phosphorothioate-modified CpG-DNAs (PS-ODN), which is a sulfur substitution for the nonbridging oxygens in the backbone providing its nuclease resistance and efficient uptake into cells, induces backbone-related side effects such as transient lymphoadenopathy, lymphoid follicle destruction, arthritis, and PS-ODN-specific IgM production [22]–[25]. Therefore, we identified the natural counterpart of the phosphodiester bond CpG-DNA (PO-ODN, MB-ODN 4531(O)) from *Mycobacterium bovis* genomic DNA to induce optimal innate immune responses without severe side effects [25], [26]. Induction of effective immune response is investigated in human and mouse cells stimulated with MB-ODN 4531(O) encapsulated in a DOPE∶CHEMS (1∶1 ratio) complex (Lipoplex(O)) [27], [28]. Furthermore, complexes of B cell epitope peptide and Lipoplex(O) without carriers significantly enhanced peptide-specific IgG production depending on TLR9 [28].

The transmembrane 4 superfamily member 5 protein (TM4SF5) has been implicated in hepatocellular carcinoma (HCC) [29]. Previously, we screened the B cell epitope of the hTM4SF5 protein and revealed the potent production of epitope-specific antibodies in mice immunized with a complex of human TM4SF5R2-3 peptide (hTM4SF5R2-3) and Lipoplex(O) [28]. We also produced the monoclonal antibody by immunization with a complex of antigenic peptide (hTM4SF5R2-3) and Lipoplex(O), which has functional effects on human HCC cells (Huh-7) expressing the antigen [28].

Here, we found that IgG production induced by a complex of B cell epitope and Lipoplex(O) without carriers is dependent on MHC, CD4^+^ cells and Th1 differentiation. In addition, we report that immunization with a complex of a specific B cell epitope of hTM4SF5 protein and Lipoplex(O) protected mice from mouse BNL 1ME A.7R.1 HCC (BNL-HCC) cell implantation. Our results may be used for prophylaxis and therapy of HCC by the development of an epitope-based peptide vaccine.

## Materials and Methods

### CpG-DNA

Natural phosphodiester bond CpG-DNA, specifically MB-ODN 4531(O), was obtained from ST Pharm Co., Ltd [26]. MB-ODN 4531 consisted of 20 bases containing three CpG motifs (underlined): AGCAGCGTTCGTGTCGGCCT.

### Synthesis of B cell epitope peptides

The B cell epitope peptide of hTM4SF5 (hTM4SF5R2-3, ^138^NRTLWDRCEAPPRV^151^) was described in earlier research [28]. The B cell epitope peptide of mouse TM4SF5 (mTM4SF5R2-3, ^137^NDTLWNLCEAPPHV^150^) was selected on the basis of its sequence homology. The peptides were produced by Peptron using an automated peptide synthesizer (Peptron III-R24, Peptron). The peptides were purified by reverse-phase HPLC (Prominence HPLC, Shimadzu Corp.) to a purity level that exceeded 95%. The peptide was identified using a mass spectrometer (HP 1100 Series LC/MSD, Hewlett-Packard).

### Preparation of B cell epitope and CpG-DNA co-encapsulated in DOPE∶CHEMS complexes

The liposomes CHEMS and DOPE were purchased from Sigma-Aldrich. Liposome complexes consisting of B cell epitope and CpG-DNA (MB-ODN 4531(O)) co-encapsulated with DOPE∶CHEMS were prepared as reported previously [28], [30]. Briefly, DOPE and CHEMS were mixed in 10% ethanol at a molar ratio of 1∶1, evaporated with nitrogen gas to make a solvent-free lipid film, and resuspended in a mixture containing equal volumes of water-soluble MB-ODN 4531(O) (50 µg/ml) and peptide (50 µg/ml), followed by vigorous stirring at room temperature for 30 min. After adjusting the pH to 7.0, the complex of peptide and Lipoplex(O) was sonicated lightly for 30 s with a sonicator (Soniifier 450, Branson Ultrasonics). After the complex was filtered with a 0.22 µm filter, the complex was freeze-thawed three times with liquid nitrogen.

### Mice and immunization

We purchased four-week-old male C57BL/6 (H-2^d^) and BALB/c (H-2^b^) mice from Central Lab. Animal, Inc., BALB/c TLR9 knockout mice from Oriental Bioservice, Inc., and BALB/c STAT4 (Stat4^tm1Gru^) and STAT6 (Stat6^tm1Gru^) knockout mice, as well as MHC class II knockout mice and OT-II transgenic mice from Jackson Laboratory. Mice were maintained under specific-pathogen-free conditions in a controlled environment (20–25°C, 32–37% humidity). All animal procedures performed in this study are in accordance with the recommendations in the Guide for the Care and Use of Laboratory Animals of the National Veterinary Research & Quarantine Service of Korea. The protocol was approved by the Institutional Animal Care and Use Committee of Hallym University (Permit Number: Hallym 2009-52). The mice were sacrificed under Zoletil 50+Rompun anesthesia, and all efforts were made to minimize suffering. On three occasions at 10 day intervals, the mice were injected intraperitoneally (i.p.) with 50 µg of peptides supplemented with 50 µg of MB-ODN4531(O) encapsulated in the DOPE∶CHEMS complex (200 µl/mouse).

### Anti-CD4 antibody treatment

Hybridoma GK1.5 (rat anti-mouse CD4 monoclonal antibody, IgG2b) was obtained from the American Type Culture Collection (ATCC). A sample of GK1.5 was produced in ascites and purified by protein A column chromatography. A 100 µg sample of GK1.5 per mouse was injected four times i.p. on days −3, −1, 1, and 3 relative to the time of immunization. Blood samples were obtained by orbital bleeding. The effective depletion of CD4^+^ cells was analyzed by FACSCalibur (Becton Dickinson) using anti-CD4 antibody, anti-CD8 antibody, and anti-CD3 antibody.

### Measurement of IL-12

BALB/c mice, BALB/c STAT4 knockout mice and STAT6 knockout mice were injected i.p. with 50 µg of hTM4SF5R2-3 peptide supplemented with 50 µg of MB-ODN4531(O) encapsulated in the DOPE∶CHEMS complex (200 µl/mouse). After 24 h, blood samples were obtained by orbital bleeding. The IL-12 amounts in the sera were then measured using commercially available ELISA kits (R&D Systems).

### Antigen-specific Ig ELISA assay

Mouse sera were achieved by orbital bleeding before each injection as well as by sacrifice 10 days after final injection. To determine the amounts and titers of total IgG, IgG1, and IgG2a, 96-well immunoplates (Nalgen Nunc International) were coated with 5 µg/ml of each peptide and then blocked with 0.05% of Tween-20 in PBS (PBST) containing 1% BSA. Total IgG, IgG1, and IgG2a levels were measured as previously described [28].

### Cross-reactivity of mouse anti-hTM4SF5 monoclonal antibody: Competitive ELISA assay

Production of mouse monoclonal antibodies to the hTM4SF5 protein and the reactivity of the monoclonal antibodies were described in earlier research [28]. We coated 96-well immunoplates with 5 µg/ml of hTM4SF5R2-3 peptide and then blocked them with PBST containing 1% BSA to obtain the titration curves of anti-TM4SF5 antibody. The monoclonal antibodies were added to the top row of each plate, and serial 1∶4 dilutions in PBST were then placed into subsequent rows. To perform a competition assay, diluted monoclonal antibodies were preincubated with the indicated amounts of mTM4SF5R2-3 peptide for 30 min and then added to the wells of each plate. The plates were incubated for 2 h at room temperature, washed with PBST, and then incubated with detecting antibody such as anti-IgG antibody conjugated with horseradish peroxidase for 2 h. A colorimetric assay was developed with a TMB substrate solution, and the absorbance at 450 nm was measured using a Spectra Max 250 microplate reader.

### Cell culture

The mouse hepatoma cell lines BNL-HCC and H2.35 were obtained from ATCC. BNL-HCC is a chemically transformed mouse liver cell line derived from the normal BALB/c embryonic liver cell line BNL CL2 [31]. The cells were cultured in DMEM medium containing 10% FBS, 25 mM HEPES, 100 U/ml of penicillin, and 100 µg/ml of streptomycin at 37°C in an atmosphere of 95% air and 5% CO_2_. H2.35 cells were cultured at 33°C in an atmosphere of 90% air and 10% CO_2_. H2.35 is a SV40 transformed mouse liver cell line derived from normal BALB/c hepatocytes [32].

### Detection of mouse TM4SF5 expression

To analyze the mTM4SF5 expression, we performed RT-PCR and FACS analysis. Total RNAs were extracted with an RNeasy Mini Kit (Qiagen), and the cDNA was generated as described previously [33]. The standard PCR reaction was performed for 25 cycles with the following primer sets: mouse GAPDH, 5′-ATGGTGAAGGTCGGTGTGAACG-3′ and 5′-GTTGTCATGGATGATCTTGGCC-3′(501 bp); mTM4SF5, 5′-CGCTTACTTGCGAAATGACA-3′ and 5′-TTTCCTGCAATCGCCACACA-3′ (174 bp). The expression of mTM4SF5 protein was confirmed by FACS analysis with the purified anti-hTM4SF5 mAb.

### MTT assay

To measure the growth of cells, an MTT assay was performed with a 3-(4,5-dimethylthiazole-2-yl)-2,5-diphenyl tetrazolium bromide (MTT, Sigma-Aldrich) solution as described previously [34]. The growth of BNL-HCC cells and H2.35 cells treated with anti-hTM4SF5 monoclonal antibody (5 µg/ml) for 5 days was determined by MTT assay as reported previously [28]. The MTT solution was added to each well at the indicated time periods and the plates were incubated for an additional 4 h at 37°C. After the removal of the medium, the formazan crystals were solubilized in DMSO. The color development was monitored by means of a spectrophotometer at 595 nm with a reference wavelength of 650 nm.

### BrdU proliferation assay

We performed BrdU proliferation assay to investigate the cell proliferation after treatment with anti-hTM4SF5 monoclonal antibody. BNL-HCC and H2.35 cells were seeded into a 96-well microplate at a density of 10^5^ cells/well and then the cells were treated with anti-hTM4SF5 monoclonal antibody (5 µg/ml) for the indicated time periods. Subsequently, the cells were fixed, washed, and incubated with primary and secondary antibodies according to the instruction of CycLex BrdU Cellular ELISA Kit (MBL International). The immune complexes were developed with a substrate solution, and we used a Spectra Max 250 microplate reader (Molecular Devices) to measure the absorbance at 450 nm.

### Hepatocellular carcinoma mouse model

Four-week-old BALB/c mice or BALB/c TLR9 knockout mice were injected i.p. with a complex of hTM4SF5R2-3 peptide (50 µg/mouse) and Lipoplex(O) three times at 10 day intervals. Ten days after the third immunization, the mice were inoculated subcutaneously in the dorsal right flank with 5×10^6^ of BNL-HCC cells in a 50% matrigel solution (HBSS/Matrigel, 1∶1 v/v, BD Biosciences) as previously described [35]. In order to evaluate the therapeutic vaccination effect of the complex of hTM4SF5R2-3 peptide and Lipoplex(O) on a mouse tumor model, BALB/c mice were inoculated subcutaneously in the dorsal right flank with 5×10^6^ of BNL-HCC cells in a 50% matrigel solution. The mice were randomly divided into four treatment groups, after the tumor size reached 5 mm in diameter. A complex of hTM4SF5R2-3 peptide (50 µg/mouse) and Lipoplex(O) was injected three times at 10 days interval into the i.p. cavity. Size of the tumor was measured 5 days interval with calipers in three dimensions, and tumor volumes were calculated as width^2^×length/2. The mice were sacrificed 60 days after tumor cell implantation, and the tumors surgically excised and weighed. Mice were sacrificed when the tumor size reached 2,000 mm^3^ or the mice lost >20% of initial body weight in accordance with the Guide for the Care and Use of Laboratory Animals of the National Veterinary Research & Quarantine Service of Korea to minimize suffering from a large tumor burden. After the vaccination, the survival rate was recorded for 100 days.

### Histology and immunohistochemistry

For histopathological examination, tumors and organs were removed and fixed in 4% buffered formalin solution, embedded in paraffin by conventional methods, and cut into 4 µm thick sections. The specimens were stained with hematoxylin and eosin. To identify the expression of TM4SF5, the specimens were stained with anti-TM4SF5 monoclonal antibodies using standard procedures.

### Statistics

Results are expressed as mean ± standard deviation. Statistical significance between two samples was performed using Student's *t* test. A *p*-value of <0.05 was taken as statistically significant. Survival analysis was performed using Kaplan-Meier method and compared by log-lank test.

## Results

### Optimization for efficient production of epitope-specific IgG with a complex of peptide epitope and Lipoplex(O)

**Figure 1:**
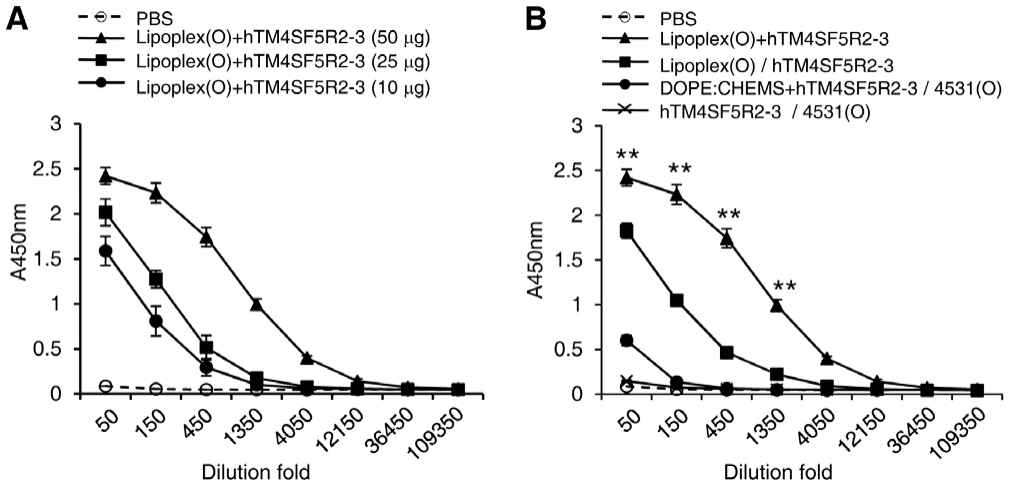
Production of IgG by immunization with a complex of hTM4SF5R2-3 peptide and Lipoplex(O). **A.** Optimal dose of peptide in the complex of hTM4SF5R2-3 peptide and Lipoplex(O). BALB/c mice (n = 3/group) were immunized with increasing amounts of hTM4SF5R2-3 peptide and Lipoplex(O) complex. **B.** Effect of the injection protocol. We immunized one group of mice with hTM4SF5R2-3 peptide after injection with Lipoplex(O) (Lipoplex(O)/TM4SF5R2-3) and another group with MB-ODN 4531(O) after injection with hTM4SF5R2-3 peptide and the DOPE∶CHEMS complex (DOPE∶CHEMS+TM4SF5/4531(O)) three times with a 10 day interval (n = 3/group). The sera were collected, and titers of the peptide-specific total IgG were assayed with an ELISA kit. These experiments were performed 3 times with similar results. **P<0.01 (*vs* PBS control).

Previously, we screened the B cell epitope of the hTM4SF5 protein and analyzed the production of IgG in the serum of BALB/c mice injected i.p. with a complex of hTM4SF5R2-3 peptide and Lipoplex(O) [28]. To determine the optimal dose of the peptide in the vaccination, we prepared complexes with increasing amounts of hTM4SF5R2-3 peptide and Lipoplex(O). When the mice were immunized with the complexes, it was noted that the production of peptide-specific IgG was enhanced in a peptide dose-dependent manner. Efficient IgG production is obtained when we used 50 µg of peptide (Figure 1A). When we used more than 50 µg of peptide, IgG production was not increased implying the presence of optimum ratio between peptide and liposome (data not shown). Next, we determined the efficient injection protocol in a vaccination with a complex of hTM4SF5R2-3 peptide and Lipoplex(O). As shown in Figure 1B, the production of IgG was efficiently induced by simultaneous immunization with a complex of the hTM4SF5R2-3 peptide and Lipoplex(O).

### Essential role of Th1 cells in IgG production induced by a peptide epitope and Lipoplex(O)

**Figure 2:**
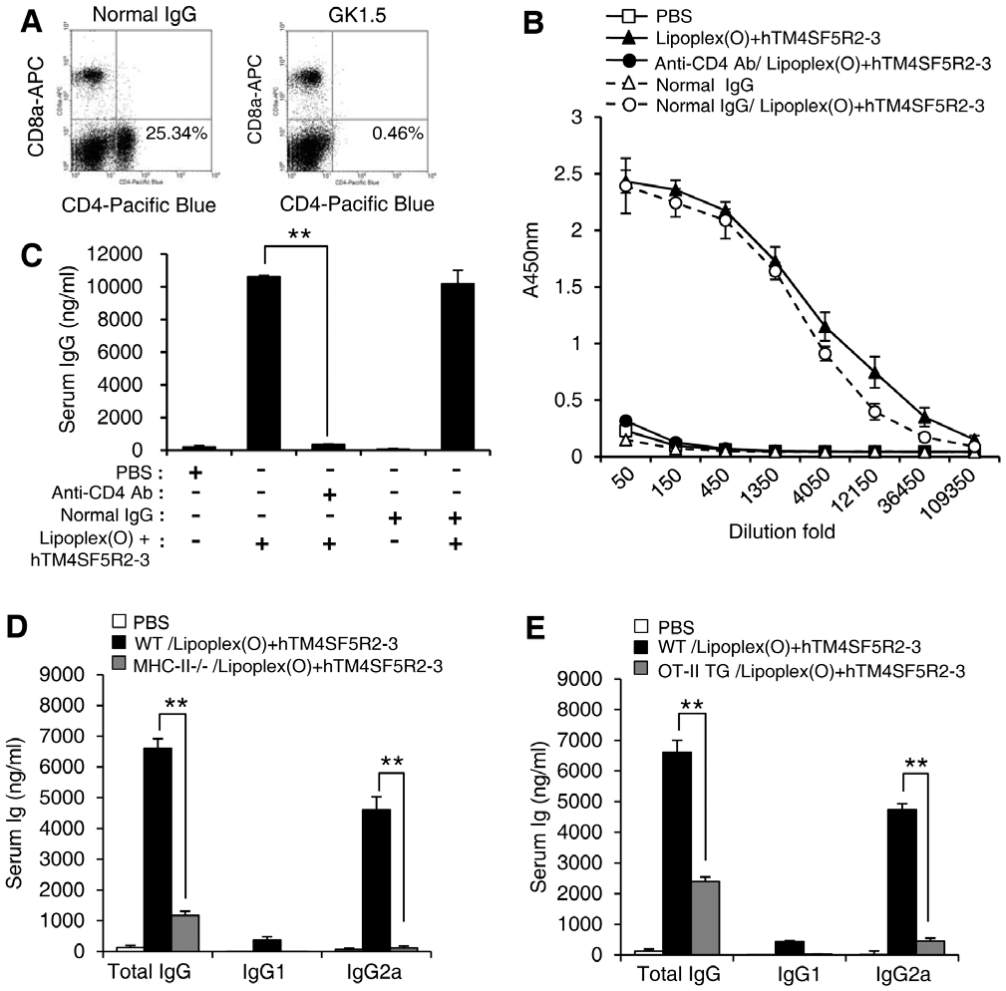
Contribution of CD4^+^ T cell on IgG production. **A–C.** Transient depletion of CD4^+^ cells prevents IgG production. GK1.5 (anti-CD4 antibody) (n = 3/group) was injected i.p to deplete CD4^+^ cells (**A**). Normal IgG was used as a control. The mice were immunized with hTM4SF5R2-3 peptide and Lipoplex(O) complex. The sera were collected, and titration curves (**B**) and amounts (**C**) of the peptide-specific total IgG were assayed with an ELISA kit. **D.** Defect in CD4^+^ T cell activation in MHC class II−/− mice prevents IgG production. C57BL/6 mice and C57BL/6 MHC class II−/− mice (n = 3/group) were injected i.p. with the hTM4SF5R2-3 peptide and Lipoplex(O) complex. **E.** Defect in specific CD4^+^ T cell activation prevents IgG production in OT-II transgenic mice. C57BL/6 mice and C57BL/6 OT-II transgenic mice (OT-II TG) (n = 3/group) were injected i.p. with the hTM4SF5R2-3 peptide and Lipoplex(O) complex. These experiments were performed 3 times with similar results. Each bar is expressed as the Mean ± SD of three mice. **P<0.01.

To understand the immune response induced by a complex of B cell epitope and Lipoplex(O) without carriers, we examined whether or not the IgG production is dependent on MHC, CD4^+^ cells and Th1 differentiation. First, we evaluated the requirement of CD4^+^ T cells. For the depletion of CD4^+^ cells, anti-mouse CD4 monoclonal antibody (GK1.5) was injected four times i.p. using a previously described method [36]. The CD4^+^-depleted mice (Figure 2A) revealed drastically reduced production of the epitope-specific IgG (Figure 2B,C). Next, we observed the involvement of MHC and TCR. The production of peptide-specific IgG and IgG2a was also reduced in MHC class II knockout mice with a CD4^+^ deficiency (Figure 2D) and in OT-II transgenic mice in which OVA-specific TCR transgenic CD4^+^ T cells are a major portion of the CD4^+^ T cell population (Figure 2E). Therefore, IgG production induced by a complex of B cell epitope and Lipoplex(O) requires CD4^+^ T cells, MHC, and TCR. The production of OVA-specific-IgG was also reduced in MHC class II knockout mice (Figure S1); OT-II transgenic mice produced OVA-specific-IgG antibodies at titers comparable to wild-type mice (Figure S2).

**Figure 3:**
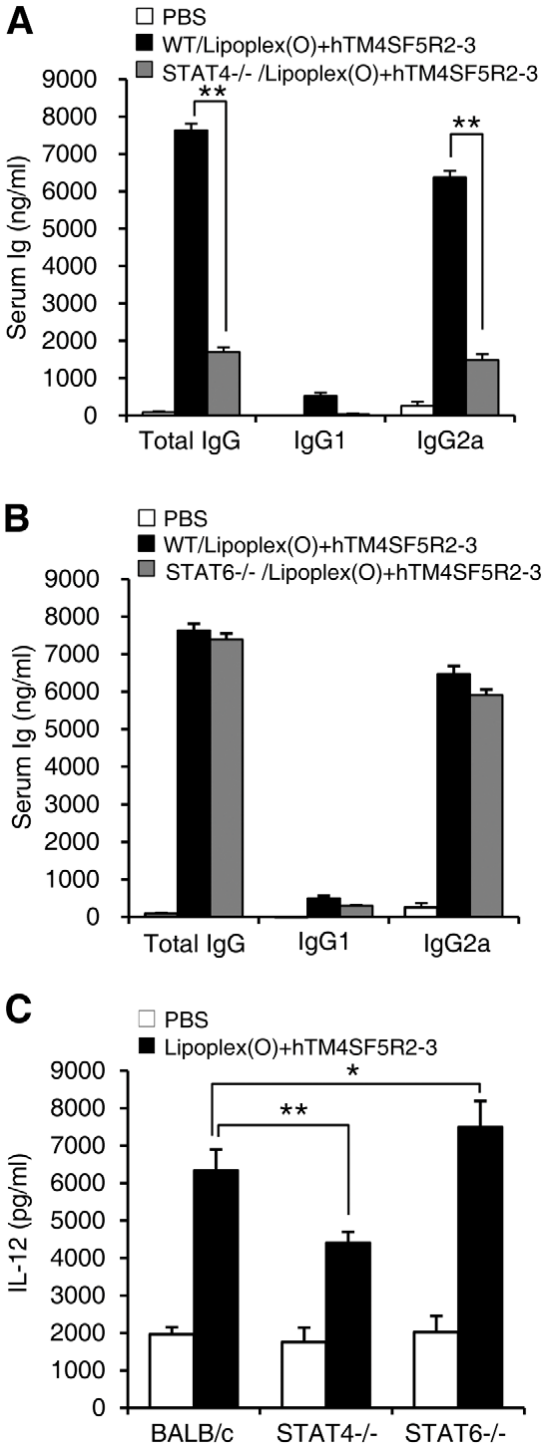
Contribution of Th1 differentiation on IgG production. STAT4 but not STAT6 is required for IgG production by a complex of peptide and Lipoplex(O). **A,B.** BALB/c mice, BALB/c STAT4−/− mice (**A**), and BALB/c STAT6−/− mice (**B**) (n = 3/group) were injected i.p. three times with a 10 day interval with the hTM4SF5R2-3 peptide and Lipoplex(O) complex. The sera were collected, and the amounts of the peptide-specific total IgG, IgG1, and IgG2a were assayed with an ELISA kit. These experiments were performed 3 times with similar results. **C.** BALB/c mice, BALB/c STAT4−/− mice, and BALB/c STAT6−/− mice (n = 5/group) were injected i.p with hTM4SF5R2-3 peptide and Lipoplex(O) complex, and sera from the mice were harvested at 24 h after injection. The levels of IL-12 in the serum were measured with an ELISA assay. Each bar is expressed as the Mean ± SD of three mice. *P<0.05, **P<0.01.

Furthermore, we examined the involvement of Th1 differentiation in peptide-specific IgG production. Previous reports showed that the Th1 responses are associated with production of IgG2a antibody, while the Th2 responses are associated with switching to the IgG1 isotype [37], [38]. STAT4 is required in the immune responses for IL-12-mediated IFN-γ production [38], [39]. Therefore, STAT4 wild type mice produced significantly higher level of IgG2a compared with STAT4 knockout mice [40]. In STAT6 knockout mice, highly significant decrease in the level of IgG1 isotype was reported [38], [41]. The fact that IgG and IgG2a production was reduced in STAT4 knockout mice reveals that, in response to immunization, STAT4 promotes the differentiation of T cells into Th1 cells (Figure 3A). However, as shown in Figure 3B, STAT6 knockout has no effect on IgG (IgG2a) production. These results suggest that Th1 cells play an essential role in IgG (IgG2a) production induced by a complex of the B cell epitope and Lipoplex(O). We also observed that the level of IL-12 induced by a complex of B cell epitope and Lipoplex(O) was decreased in the sera of STAT4 knockout mice compared with the wild type mice. However, STAT6 knockout mice produced higher amounts of IL-12 by a complex of B cell epitope and Lipoplex(O) compared with the wild type mice (Figure 3C) as reported previously [42], [43].

### Effect of anti-hTM4SF5 monoclonal antibody on mouse hepatocellular carcinoma cells *in vitro*

**Figure 4:**
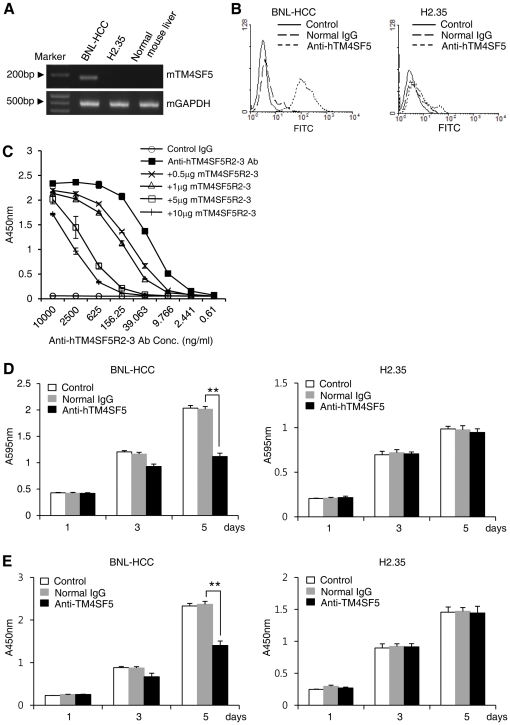
Effects of anti-hTM4SF5 monoclonal antibody on mouse HCC cells. **A.** The expression levels of mTM4SF5 mRNA in the indicated mouse HCC cell lines and normal mouse hepatocytes were analyzed by RT-PCR. **B.** Detection of mTM4SF5 in BNL-HCC cells and H2.35 cells by anti-hTM4SF5R2-3 peptide monoclonal antibody according to a FACS analysis. Normal mouse IgG was used as a control. **C.** Reactivity of anti-hTM4SF5R2-3 monoclonal antibody with the mTM4SF5R2-3 peptide. The hTM4SF5R2-3 peptide was immobilized on a plate, and competitive ELISA was performed using increasing amounts of soluble mTM4SF5R2-3 peptide. **D.** Effect of anti-hTM4SF5R2-3 peptide monoclonal antibody on the growth of BNL-HCC cells and H2.35 cells. Cell growth was measured by an MTT assay. **E.** Effect of anti-hTM4SF5R2-3 peptide monoclonal antibody on the proliferation of BNL-HCC cells and H2.35 cells. The DNA synthesis activity was monitored by BrdU incorporation assay. Each bar is expressed as the Mean ± SD of three experiments. **P<0.01.

Previously, we evaluated the functional effects of anti-hTM4SF5R2-3-specific monoclonal antibody produced by immunization with a complex of hTM4SF5R2-3 peptide and Lipoplex(O) on antibody-mediated hTM4SF5 targeting in human HCC cells [28]. To evaluate the prophylactic efficacy of a vaccine containing the complex of hTM4SF5R2-3 peptide and Lipoplex(O) on a HCC mouse model, we examined the functional effects of anti-hTM4SF5R2-3 monoclonal antibody on mouse HCC cells. Clustal W analysis revealed that hTM4SF5 and mTM4SF5 share 82% similarity (Figure S3). We first identified the expression of mTM4SF5 and confirmed the functional effects of anti-hTM4SF5R2-3 peptide monoclonal antibody in mouse HCC cells. The expression of mTM4SF5 was detected in the BNL-HCC cell line at the mRNA level (Figure 4A). The anti-hTM4SF5R2-3 peptide monoclonal antibody was bound to BNL-HCC cells, demonstrating cross-reactivity of anti-hTM4SF5R2-3 peptide monoclonal antibody to mTM4SF5 protein (Figure 4B). To confirm that anti-hTM4SF5R2-3 peptide monoclonal antibody targets the mTM4SF5 protein, we used an ELISA test which determined that anti-hTM4SF5R2-3 peptide monoclonal antibody reacted with the hTM4SF5R2-3 peptide as well as with the mTM4SF5R2-3 peptide (Figure 4C). To consider the effect of the anti-hTM4SF5R2-3 peptide monoclonal antibody on mouse HCC cell growth, we performed an MTT assay. Treatment with the anti-hTM4SF5R2-3 peptide monoclonal antibody delayed the growth of BNL-HCC cells. In contrast, there was no effect on the growth of H2.35 cells which did not express mTM4SF5 (Figure 4D). We then investigated the effects of the anti-hTM4SF5R2-3 peptide antibody on DNA synthesis using the BrdU incorporation assay. The rate of DNA synthesis in BNL-HCC cells treated with anti-hTM4SF5R2-3 peptide monoclonal antibody was reduced in five days by approximately 40% compared to normal IgG treated-BNL-HCC cells. However, there was no significant reduction in the rate of DNA synthesis of H2.35 cells (Figure 4E).

### Prophylactic efficacy of a vaccine containing a hTM4SF5 epitope and Lipoplex(O) complex against tumor formation by hepatocellular carcinoma cells implanted in mice

**Figure 5:**
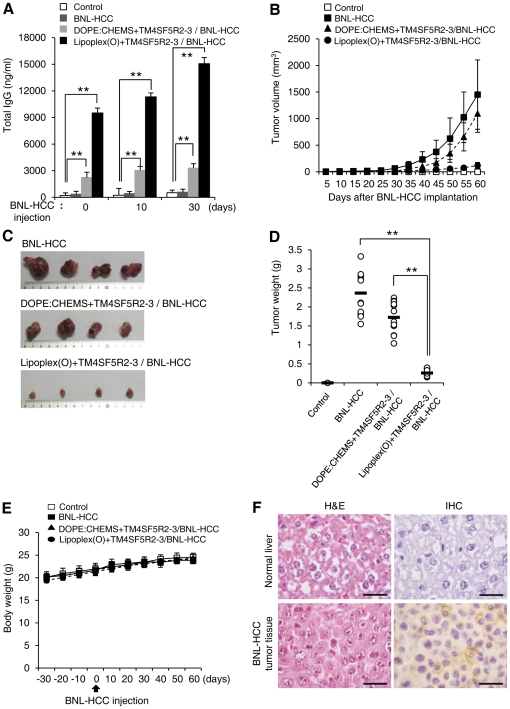
Prophylactic efficacy of a vaccine containing hTM4SF5R2-3 peptide and Lipoplex(O) complex in HCC implanted mouse. BALB/c mice were immunized with a complex of hTM4SF5R2-3 peptide and Lipoplex(O). The immunized mice were implanted with the BNL-HCC cells (n = 12 per group; n = 10 per PBS-treated control). **A.** Induction of a strong serologic response to BNL-HCC cell implantation by vaccination with epitope and Lipoplex(O). The sera were collected, and the amounts of mTM4SF5R2-3 peptide-specific total IgG were assayed using an ELISA kit. Each bar is expressed as the Mean ± SD of 10 or 12 mice. **B–D.** Tumor formation in mice implanted with BNL-HCC cells was inhibited by vaccination with the hTM4SF5R2-3 peptide and Lipoplex(O) complex 60 days after the implantation of BNL-HCC cells. Tumor volumes were calculated as (length×width^2^)/2 (**B**). Macroscopic appearance of HCC tumor tissues (**C**). Tumor growth was measured by tumor weight (**D**). **E.** Body weights were measured at the indicated time intervals. **F.** Histology of normal liver and tumor tissue derived from BNL-HCC cell-implanted mice was observed by staining with hematoxylin and eosin (H&E, left panel). An immunohistochemical analysis (IHC) was performed with anti-hTM4SF5R2-3 monoclonal antibody (right panel). TM4SF5 positive area was expressed as brown color. Scale bars, 500 µm. **P<0.01.

**Figure 6:**
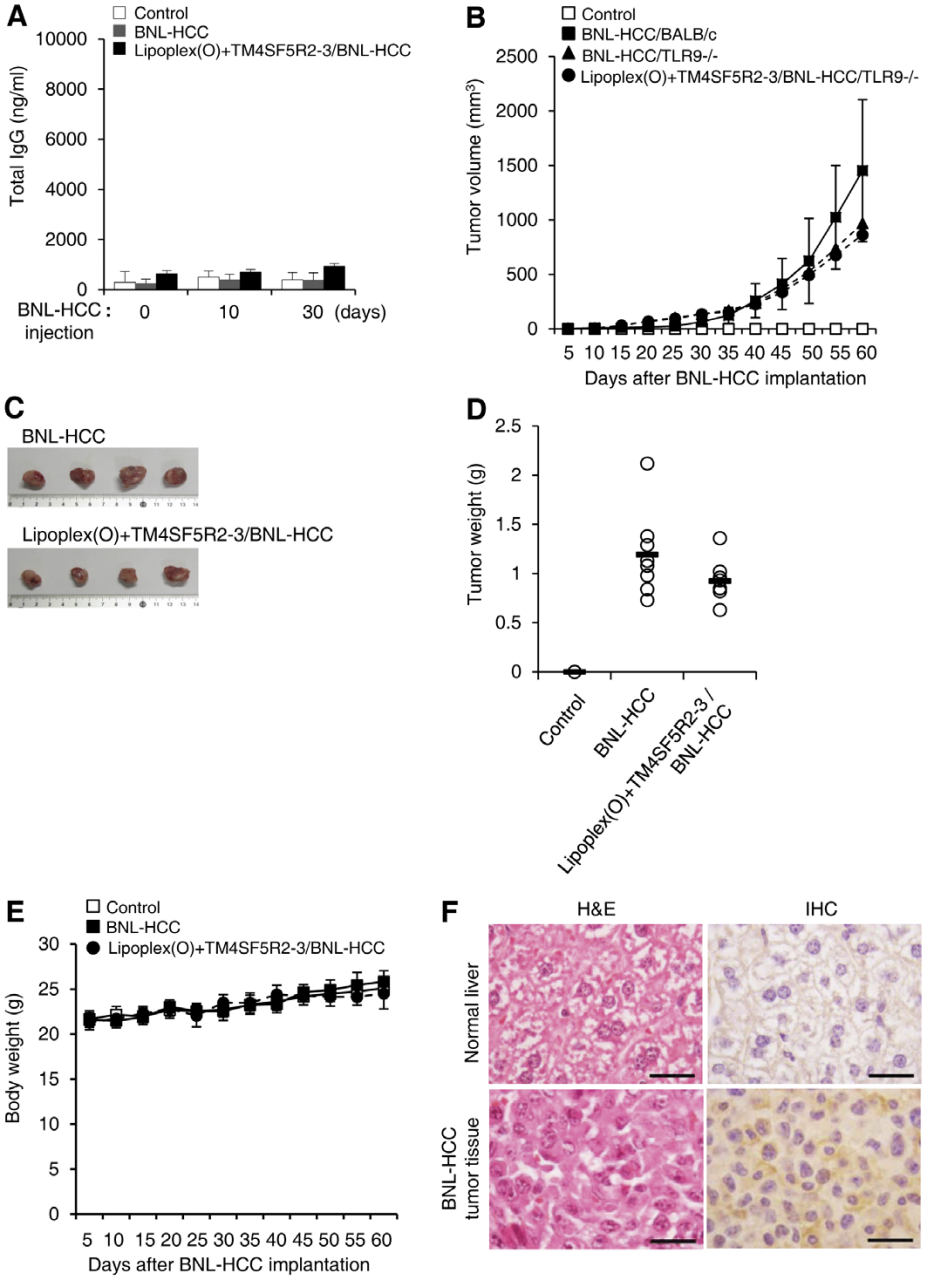
Involvement of TLR9 on prophylactic efficacy of a vaccine containing hTM4SF5R2-3 peptide and Lipoplex(O) complex. BALB/c TLR9−/− mice were immunized with a complex of hTM4SF5R2-3 peptide and Lipoplex(O) and the mice were implanted with the BNL-HCC cells (n = 8 per group). **A.** Effect of TLR9 on serologic response to BNL-HCC cell implantation after vaccination with epitope and Lipoplex(O). The sera were collected, and the amounts of mTM4SF5R2-3 peptide-specific total IgG were assayed using an ELISA kit. Each bar is expressed as the Mean ± SD of 8 mice. **B.** Tumor volumes were calculated as width^2^×length/2. The tumor volumes in TLR9−/− mice were compared with the tumor volumes in wild type BALB/c mice in Figure 5B. **C.** Macroscopic appearance of HCC tumor tissues. **D.** Tumor growth was measured by tumor weight. **E.** Body weights were measured at the indicated time intervals. **F.** Histology of normal liver and tumor tissue derived from BNL-HCC cell-implanted mice was observed by staining with hematoxylin and eosin (left panel). An immunohistochemical analysis was performed with anti-hTM4SF5R2-3 monoclonal antibody (right panel). TM4SF5 positive area was expressed as brown color. Scale bars, 500 µm.

To evaluate the prophylactic efficacy of the vaccine containing the complex of hTM4SF5R2-3 peptide and Lipoplex(O) on tumor formation, we implanted BNL-HCC cells into BALB/c mice 10 days after vaccination three times. As a negative control, two groups of mice were immunized with PBS or hTM4SF5R2-3 peptide encapsulated in DOPE∶CHEMS, followed by implantation with BNL-HCC cells. We observed significant production of hTM4SF5R2-3 peptide-specific antibody in response to implantation in the mice immunized with the complex of hTM4SF5R2-3 peptide and Lipoplex(O) (Figure 5A). BNL-HCC cells implanted into BALB/c mice grew continuously into a tumor mass, whereas tumor development was significantly inhibited in the vaccinated mice (Figure 5B–D). Vaccination with DOPE∶CHEMS and TM4SF5R2-3 induced production of hTM4SF5R2-3 peptide-specific antibody at a low level and showed weak prophylactic activity. Vaccination did not affect the body weight of the mice during the experiment, suggesting that no significant side effects were provoked by immunization with the complex of hTM4SF5R2-3 peptide and Lipoplex(O) (Figure 5E). The expression of TM4SF5 in HCC tumor tissue was confirmed by immunohistochemistry; specifically, the tumor cells were intensively immunostained by anti-hTM4SF5R2-3 peptide monoclonal antibody in the cell membrane and cytosol (Figure 5F). However, there was no significant prevention of tumor development in the TLR9−/− mice (Figure 6), which are defective in IgG production (Figure 6A), after immunization with the complex of hTM4SF5R2-3 peptide and Lipoplex(O) [28]. Interestingly, the tumor growth was lower in the TLR9−/− mice than in wild type mice (Figure 5B–D vs Figure 6B–D). We conclude that a complex of hTM4SF5R2-3 peptide and Lipoplex(O) was effective in eliciting a protective immune response via the generation of specific antibodies that bind to TM4SF5 protein and inhibit tumor growth *in vivo*.

### Therapeutic efficacy of vaccine containing a hTM4SF5 epitope and Lipoplex(O) complex on the growth of hepatocellular carcinomas in mice

**Figure 7:**
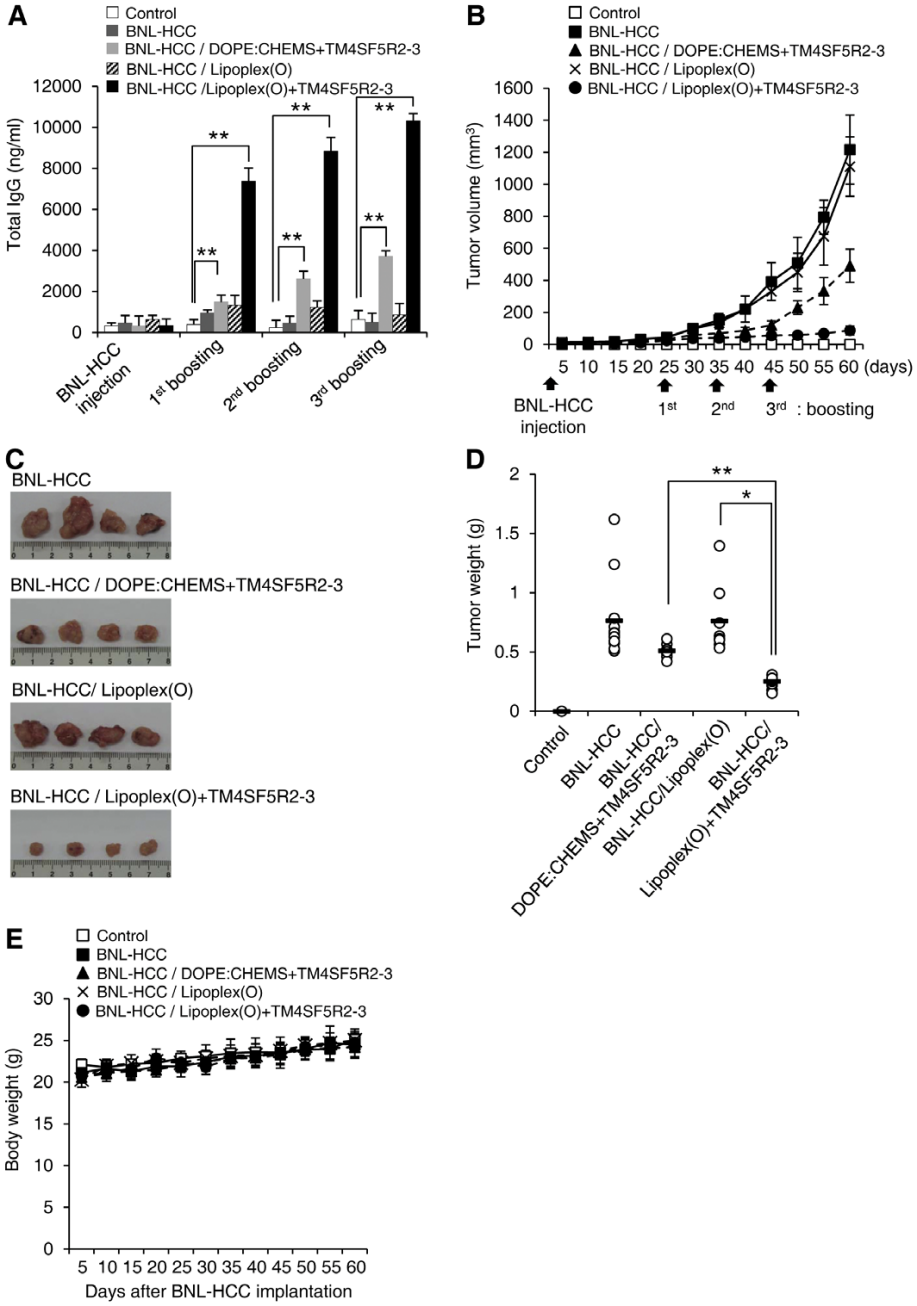
Therapeutic efficacy of a vaccine containing hTM4SF5R2-3 peptide and Lipoplex(O) complex in HCC implanted mouse. **A.** Induction of a strong serologic response by vaccination with the hTM4SF5R2-3 peptide and Lipoplex(O) complex or indicated combinations after BNL-HCC cell implantation in mice (n = 12 per hTM4SF5R2-3 peptide+Lipoplex(O) group; n = 8 per PBS-treated control group, Lipoplex(O) group, and DOPE∶CHEMS+hTM4SF5R2-3 peptide group). The sera were collected 10 days after vaccination, and the amounts of mTM4SF5R2-3 peptide-specific total IgG were assayed using an ELISA kit. Each bar is expressed as the Mean ± SD of 8 or 12 mice. **B–D.** Inhibition of tumor formation in mice vaccinated with the hTM4SF5R2-3 peptide and Lipoplex(O) complex 60 days after the implantation of BNL-HCC cells. Tumor volumes were calculated as width^2^×length/2 (**B**). Macroscopic appearance of HCC tumor tissues (**C**). Tumor growth was measured by tumor weight of the surviving mice (**D**). **E.** Body weights were measured at the indicated time intervals. Each bar represents the Mean ± SD of 8 or 12 mice. *P<0.05, **P<0.01.

**Figure 8:**
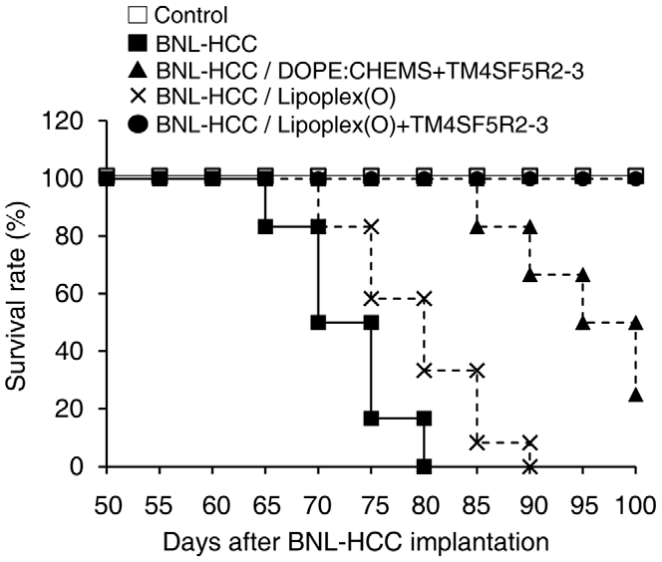
Survival rate of tumor-bearing mice. BALB/c mice were immunized with the hTM4SF5R2-3 peptide and Lipoplex(O) complex or indicated combinations after BNL-HCC cell implantation in mice (n = 12 per group; n = 10 per PBS-treated control). After the vaccination, the survival rate was recorded for 100 days.

To evaluate the therapeutic antitumor effects in tumor-bearing mice after vaccination with a complex of hTM4SF5R2-3 peptide and Lipoplex(O), we implanted BALB/c mice with BNL-HCC cells and then vaccinated them with a complex of hTM4SF5R2-3 peptide and Lipoplex(O) after 25 days. Mice vaccinated with the complex of hTM4SF5R2-3 peptide and Lipoplex(O) produced significantly higher amounts of hTM4SF5R2-3 peptide-specific antibody (Figure 7A), and significantly reduced tumor growth (Figure 7B–D) compared to the other controls. PBS-treated mice started to die 65 days after BLN-HCC implantation, with 100% of them expiring by 80 days. However, the mice vaccinated with the complex of hTM4SF5R2-3 peptide and Lipoplex(O) demonstrated prolonged survival up to 100 days post-tumor implantation (Figure 8). Thus, our data indicate that vaccination with a complex of hTM4SF5R2-3 peptide and Lipoplex(O) in HCC-bearing mice has therapeutic antitumor effects.

## Discussion

Several investigators have reported that CpG-DNA has Th1-responsive immunoadjuvant effects and that its potent immunoadjuvant effects are augmented by liposome encapsulation [5], [12], [15], [16], [21]. Suzuki *et al.* showed that CpG-DNA encapsulated in cationic liposomes induces the expression of IL-12 and IFN-γ and that CpG-DNA-liposome co-encapsulated with ovalbumin (OVA) caused the induction of OVA-specific CTLs, which exhibited potent cytotoxicity against OVA-expressing tumor [21]. In addition, SSCL improves the uptake by B cells, dendritic cells, and macrophages, and the co-encapsulation of CpG-DNA with OVA magnified Ag-specific IFN-γ and IgG production [15]. Furthermore, Li *et al.* found that CpG-DNA and HER-2/neu-derived peptide co-encapsulated in DSPC/Chol liposomes enhances the CTL response and IgG production [12]. Previously, we formulated an efficacious peptide vaccine without carriers using the natural phosphodiester bond CpG-DNA (MB-ODN 4531(O)) and a special liposome (DOPE∶CHEMS) complex (Lipoplex(O)) to improve the efficacy of peptide vaccines. We showed that immunization of BALB/c mice with a complex consisting of B cell epitope peptide and Lipoplex(O) resulted in a markedly greater abundance of epitope-specific IgG than that in mice immunized with B cell epitope peptide and MB-ODN 4531(O) co-encapsulated in other liposomes [28]. In this report, we presented the optimal dose of the peptide and an efficient injection protocol in vaccinations with a complex of hTM4SF5R2-3 peptide and Lipoplex(O). Efficient IgG production was obtained with 50 µg of peptide and simultaneous immunization with the hTM4SF5R2-3 peptide and Lipoplex(O) complex (Figure 1).

We also observed that the production of antibodies by immunization with a complex of B cell epitope peptide and Lipoplex(O) required MHC, CD4^+^ cells and Th1 differentiation (Figure 2). Although the Th1 immune response was essential for peptide-specific IgG production mediated by Lipoplex(O), the mechanism that induces the peptide-specific Th1 response remains to be investigated in detail. Further study is also needed to investigate whether or not IgG production induced by a complex of B cell epitope and Lipoplex(O) is dependent on the MHC type and if a novel mechanism is involved.

Several uniquely over-expressed antigens, including TM4SF5 [29] and glypican-3 [44], have been linked to the pathogenesis of HCC. Recently, glypican-3-derived peptide vaccine was investigated to determine its ability to induce HCC-specific CTLs [45]. The mRNA expression of TM4SF5 in human cancer is prevalently observed in pancreatic cancer, soft tissue csarcoma, gastric cancer, carcinoma of the papilla vateri, and colon cancer [46]. When the TM4SF5 overexpression in human HCC specimens was examined by immunohistochemical staining, the TM4SF5 was detected in 7 of 9 HCC tissues in the cell membrane and cytosol whereas normal liver tissues were not positive for TM4SF5 expression [29]. Therefore, TM4SF5 is presumed to be a novel molecular target for the clinical development of a type of HCC immunotherapy [47], [48]. Here, we analyzed whether the monoclonal antibody produced by immunization with a complex of hTM4SF5R2-3 peptide and Lipoplex(O) can detect TM4SF5 in tumor tissues derived from a HCC mouse model (Figure 5F and 6F).

Lee et al. reported that TM4SF5 is important in HCC formation by inducing morphological elongation, epithelial-mesenchymal transitions, abnormal cell growth *in vitro*, and tumor formation *in vivo* [29]. Overexpression of TM4SF5 in HCC cells leads to S phase progression by shortening the G1 phase period and the cells continuously proliferate under confluent conditions. The overexpression of TM4SF5 also enhances the cytosolic p27^kip1^ stability and inactivates the RhoA pathway, resulting in loss of contact inhibition through actin reorganization [29]. Activation of c-Jun N-terminal kinase (JNK) pathway associates with the ser10 phosphorylation and cytosolic localization of p27^kip1^ in the TM4SF5-expressing cells [49]. Furthermore, physical association between TM4SF5 and integrin is also capable of modulating the signaling properties of integrins [50]. Previously, we showed that the monoclonal antibody produced by immunization with a complex of hTM4SF5R2-3 peptide and Lipoplex(O) had functional effects on human HCC cell line (Huh-7 cells) expressing the antigen. The anti-hTM4SF5R2-3 peptide-specific antibody can detect native protein and induce functional changes (delay of the cell growth) in Huh-7 cells, which suggest a possible application in therapeutics [28]. Here, we investigated further that growth of mouse HCC cell line (BNL-HCC) expressing hTM4SF5 was markedly delayed by the antibody treatment but not in H2.35 cells which did not express hTM4SF5 (Figure 4). However, further study is needed to investigate the functional role of anti-hTM4SF5R2-3 monoclonal antibody in TM4SF5-mediated cell growth, which may support the future application of the antibody in HCC therapeutics. In agreement with the *in vitro* data, humoral immune responses elicited by active immunization with a complex of hTM4SF5R2-3 peptide and Lipoplex(O) prevented tumor development in a HCC mouse model (Figure 5). We also found that a complex of TM4SF5R2-3 peptide and Lipoplex(O) in HCC-bearing mice can reduce HCC tumor growth even after tumor formation (Figure 7). This is therefore a therapeutic HCC vaccine candidate.

TLR9 recognizes bacterial DNA and CpG-DNA as non-self DNA in response to bacterial invasion. The activation of TLR9 induces a strong Th1 immune response with upregulation of Th1 cytokines, costimulatory molecules and MHC molecules [17]. The enhanced Th1 responses by IL-12 and IFN-γ production contribute to the protection of the host from infectious pathogens [51], [52]. The antiviral activity against the DNA virus murine CMV *in vivo* was diminished in TLR9 knockout mice [53]. Furthermore, TLR9 knockout mice had reduced ability to clear the Gram-positive bacterial pathogen *Streptococcus pneumoniae* and Gram-negative bacterial pathogen *Klebsiella pneumoniae* [54], [55]. Interestingly, we observed that the HCC tumor growth was lower in the TLR9−/− mice than in wild type mice (Figure 6). As the relevance of TLR9 to the tumor formation is not known yet, further study is needed to clarify this issue.

Our overall results show that IgG production induced by a complex of B cell epitope and Lipoplex(O) without carriers is dependent on MHC, CD4^+^ cells and Th1 differentiation. Furthermore, this study demonstrates that vaccination with a complex of hTM4SF5R2-3 peptide and Lipoplex(O) can reduce a hepatocellular carcinoma mass in the HCC mouse model. Therefore, our strategy involving the use of the hTM4SF5R2-3 peptide may provide a novel prophylaxis measure and therapy for HCC patients with TM4SF5-positive tumors.

## Supporting Information

Figure S1 **MHC class II is required for IgG production (data correspond to** **Figure 2D** **).** C57BL/6 mice (WT) and C57BL/6 MHC class knockout mice (MHC-II−/−) (n = 3/group) were immunized with an OVA and IFA mixture. The sera were collected, and titers of OVA-specific total IgG, IgG1, and IgG2a were assayed with an ELISA kit. These experiments were performed 3 times with similar results. **P<0.01.
(TIF)

Figure S2 **T cell activation is required for IgG production (data correspond to** **Figure 2E** **).** C57BL/6 mice and C57BL/6 OT-II transgenic mice (OT-II TG) (n = 3/group) were immunized with an OVA and IFA mixture. The sera were collected, and titers of OVA-specific total IgG were assayed with an ELISA kit. These experiments were performed 3 times with similar results.
(TIF)

Figure S3 **CLUSTAL alignment of hTM4SF5 and mTM4SF5.** The location of the TM4SF5R2-3 epitopes is shown.
(TIF)
