# Supplementary figures and images for: Prevention and Therapy of Hepatocellular Carcinoma by Vaccination with TM4SF5 Epitope-CpG-DNA-Liposome Complex without Carriers
Source: PLoS One. 2012 Mar 12;7(3):e33121. doi: 10.1371/journal.pone.0033121 (PMC3299740; doi:10.1371/journal.pone.0033121)

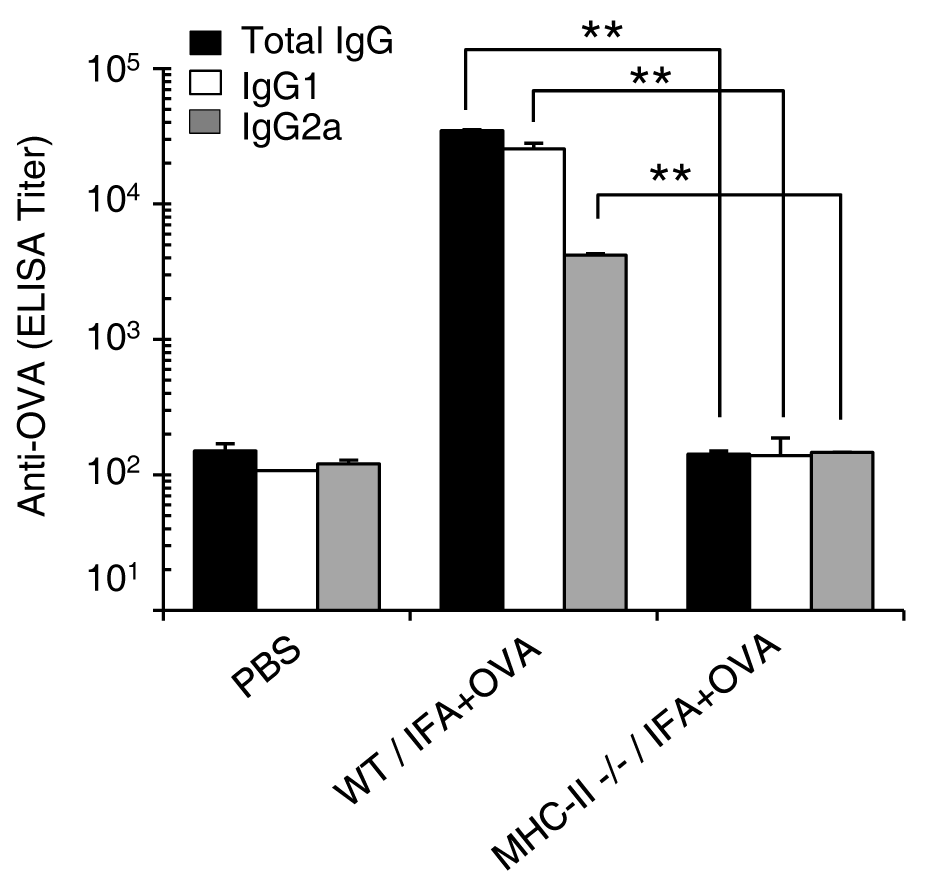

Supplement: Figure S1 — MHC class II is required for IgG production (data correspond to Figure 2D ). C57BL/6 mice (WT) and C57BL/6 MHC class knockout mice (MHC-II−/−) (n = 3/group) were immunized with an OVA and IFA mixture. The sera were collected, and titers of OVA-specific total IgG, IgG1, and IgG2a were assayed with an ELISA kit. These experiments were performed 3 times with similar results. **P<0.01. (TIF) [file pone.0033121.s001.tif]

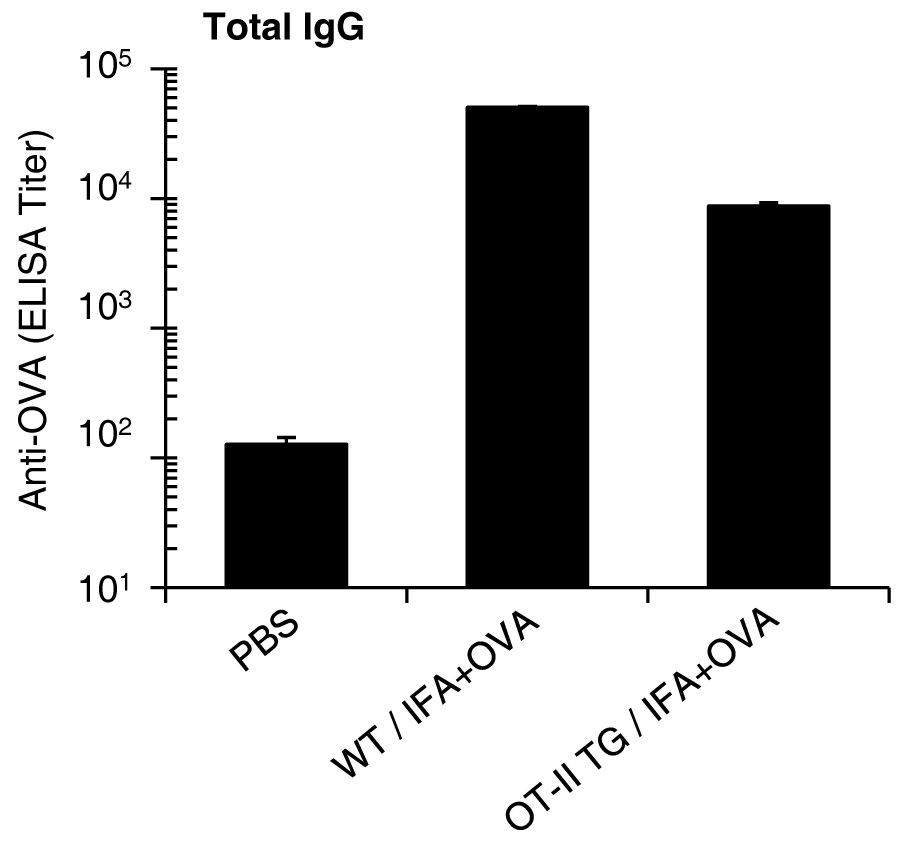

Supplement: Figure S2 — T cell activation is required for IgG production (data correspond to Figure 2E ). C57BL/6 mice and C57BL/6 OT-II transgenic mice (OT-II TG) (n = 3/group) were immunized with an OVA and IFA mixture. The sera were collected, and titers of OVA-specific total IgG were assayed with an ELISA kit. These experiments were performed 3 times with similar results. (TIF) [file pone.0033121.s002.tif]

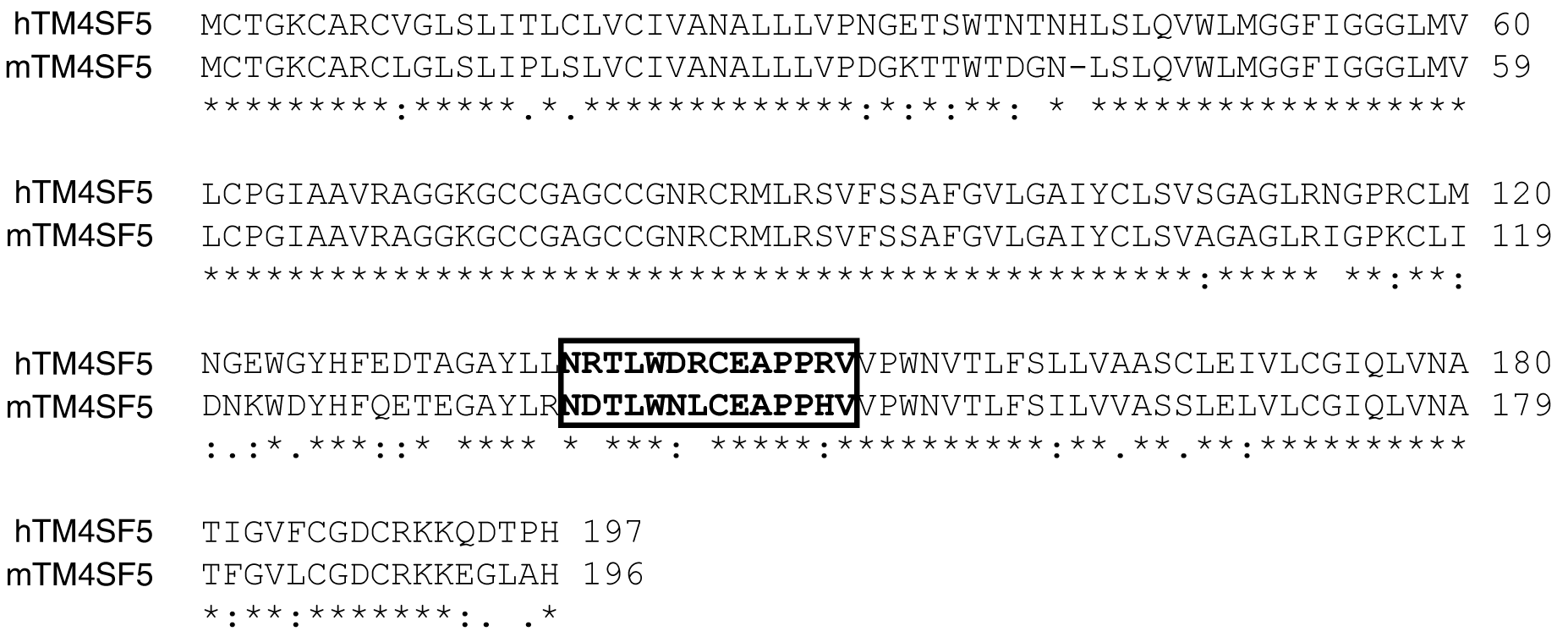

Supplement: Figure S3 — CLUSTAL alignment of hTM4SF5 and mTM4SF5. The location of the TM4SF5R2-3 epitopes is shown. (TIF) [file pone.0033121.s003.tif]
